# Modulation of the Interactions Between α-Synuclein and Lipid Membranes by Post-translational Modifications

**DOI:** 10.3389/fneur.2021.661117

**Published:** 2021-07-15

**Authors:** Rosie Bell, Michele Vendruscolo

**Affiliations:** Centre for Misfolding Disease, Department of Chemistry, University of Cambridge, Cambridge, United Kingdom

**Keywords:** Parkinson's disease, protein aggregation, lipid membranes, post-translational modifications, lipid homeostasis, protein homeostais

## Abstract

Parkinson's disease is characterised by the presence in brain tissue of aberrant inclusions known as Lewy bodies and Lewy neurites, which are deposits composed by α-synuclein and a variety of other cellular components, including in particular lipid membranes. The dysregulation of the balance between lipid homeostasis and α-synuclein homeostasis is therefore likely to be closely involved in the onset and progression of Parkinson's disease and related synucleinopathies. As our understanding of this balance is increasing, we describe recent advances in the characterisation of the role of post-translational modifications in modulating the interactions of α-synuclein with lipid membranes. We then discuss the impact of these advances on the development of novel diagnostic and therapeutic tools for synucleinopathies.

## Introduction

### Physiological Functions of α-Synuclein That Involve the Binding to Lipid Membranes

**Figure 1:**
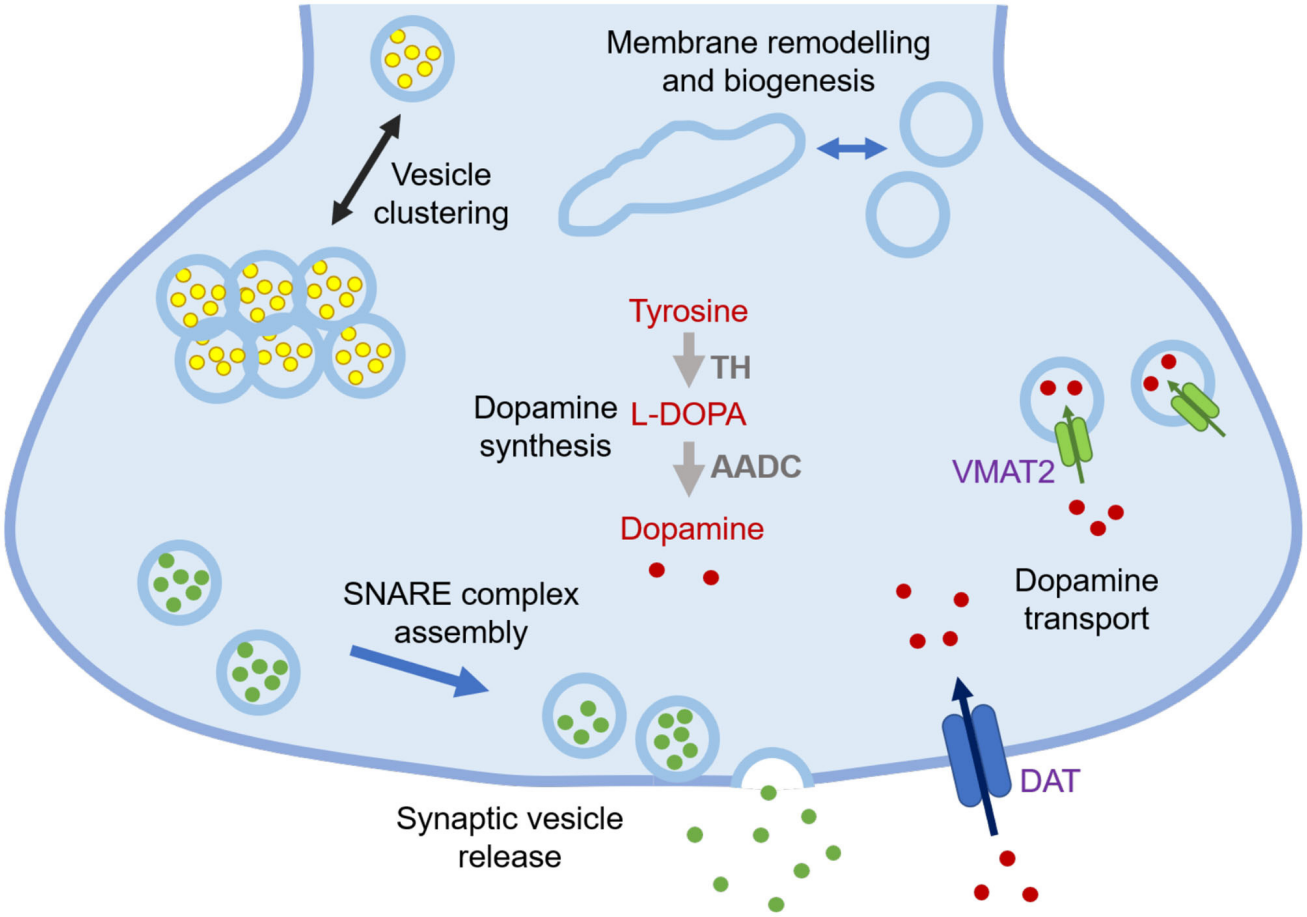
Schematic illustration of currently known functions of α-synuclein at synapses. The functions of α-synuclein are still to be fully elucidated, but at synapses they may include lipid-related functions, such as regulation of synaptic vesicle pools via sorting and clustering, remodelling of lipid membranes, in particular tubulation and biogenesis, and promotion of SNARE complex assembly and synaptic vesicle release, as well as non-lipid related functions, including modulation of dopamine synthesis via tyrosine hydroxylase (TH) that converts tyrosine into L-3,4-dihydroxyphenylalanine (L-DOPA) and regulation of dopamine transport through dopamine transporter (DAT) and vesicular monoamine transporter (VMAT2) (7, 8).

α-Synuclein is a 140-residue protein encoded by the *SCNA* gene and comprised of three domains, an N-terminal amphipathic region (residues 1-60), a central aggregation-prone region (residues 61-95) known as the non-Aβ component (NAC), and a negatively charged C-terminal domain (residues 96-140) (1). In the cellular environment, α-synuclein can populate different states, including a disordered monomer in solution (2–4), and a partially structured α-helical state at the surface of lipid membranes (5, 6). Many of the synaptic functions that have been proposed for α-synuclein involve its association with cell membranes, including regulation of synaptic plasticity, dopamine levels, and synaptic vesicle trafficking (Figure 1) (7, 9).

The regulation of synaptic vesicles by α-synuclein may include the coordination of their availability for neurotransmitter release by binding, ordering, localizing, and sequestering them (10). The N-terminal amphipathic domain is crucial for the interaction of α-synuclein with lipid membranes. Upon interacting with highly curved lipid membranes, the N-terminal and NAC regions can form an antiparallel broken α-helical conformation, with the two α-helices comprising residues 1-37 and 45-94, respectively (11). In the presence of lipid membranes with lower curvature, the two α-helices can merge into a continuous one (12). A more recent study has shown that upon binding to small unilamellar lipid vesicles (SUVs) the first 25 residues of α-synuclein tend to be anchored to the vesicle surfaces, while the central region (residues 26-98) can transiently bind other vesicles in a “double anchor” mechanism, with the C-terminal residues remaining disordered (5, 13).

Cell membranes exhibit complex and heterogeneous compositions, with the prime constituents being phospholipids (14), which are molecules composed of a glycerol head group esterified by two fatty acid chains with varying degrees of unsaturation (14, 15). The most abundant phospholipids in the outer leaflets of cell membranes are phosphatidic acid (PA), phosphatidylcholine (PC), phosphatidylethanolamine (PE), sphingomyelin (SM), and cholesterol (14), while in the inner leaflets phosphatidylinositol (PI), and phosphatidylserine (PS) are most abundant (10). The composition of phospholipids in lipid membranes alters their interactions with proteins (16), and in particular the acidic non-bulky head groups of phospholipids tend to attract the positively charged N-terminus of α-synuclein (17).

### α-Synuclein in Parkinson's Disease

Parkinson's disease is the most prevalent neurodegenerative movement disorder, effecting over 6 million individuals worldwide (18, 19). This disease is characterised by a loss of dopaminergic neurons in the *substantia nigra* and by the presence of Lewy body and Lewy neurites, which are deposits containing α-synuclein (20) as well as a variety of other cellular components (21, 22). Mutations within the *SNCA* gene (A53T/E, A30P, E46K, H50Q, and G51D) have linked α-synuclein to familial Parkinson's disease and related synucleinopathies (23–28). In addition, *SNCA* duplication and triplication were also found to promote the early onset form of the disease (29–31).

These considerations firmly link α-synuclein to Parkinson's disease (8, 32–34), in particular through its interactions with lipid membranes (15, 35). This protein thus represents a primary therapeutic target for this disease and related synucleinopathies (36–41).

### Aggregation of α-Synuclein

**Figure 2:**
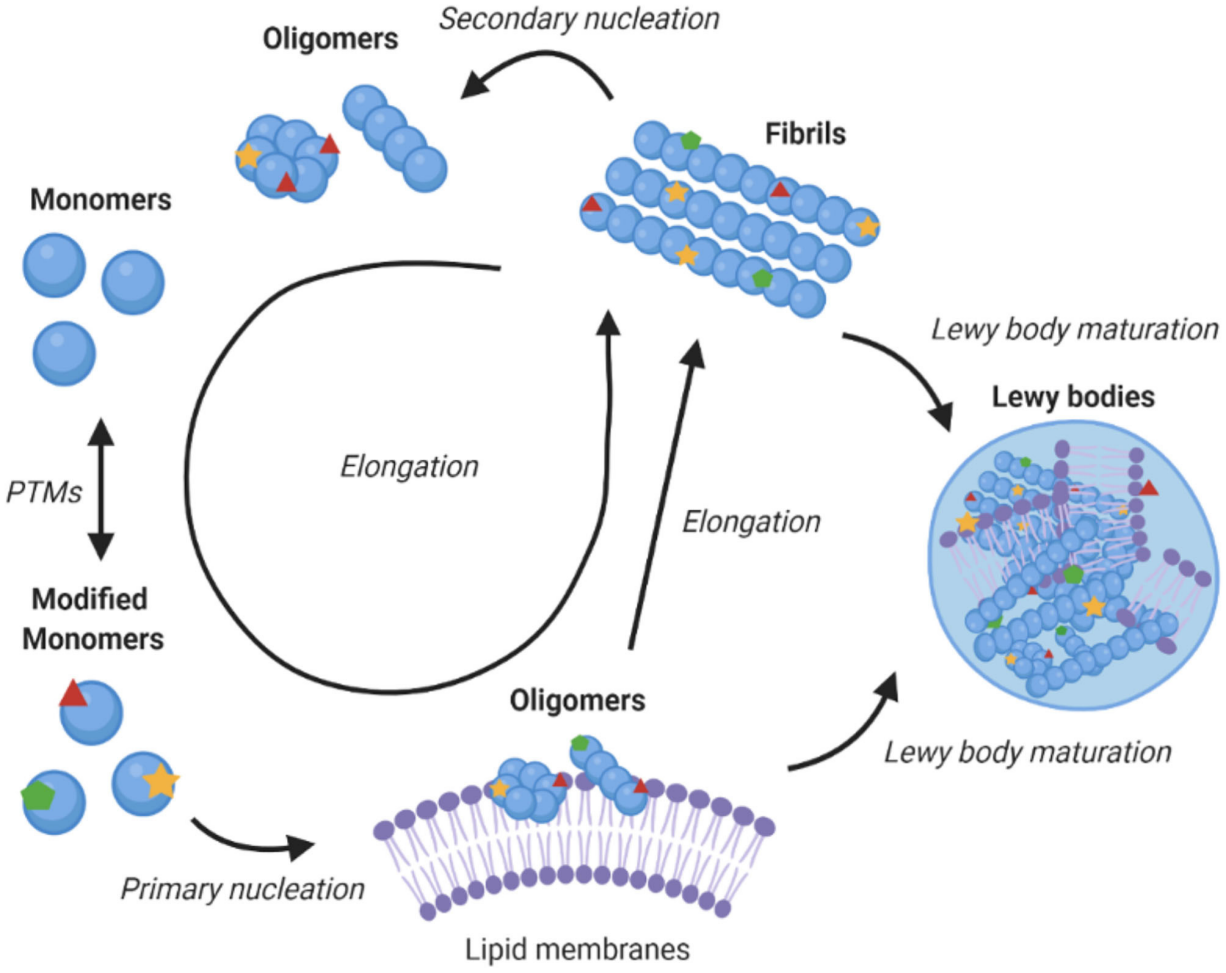
Role of lipid membranes in α-synuclein aggregation and its links with Parkinson's disease. The figure shows a schematic illustration of a kinetic network model for the aggregation of α-synuclein into amyloid fibrils. Initially, α-synuclein monomers combine to form oligomers, in a heterogeneous nucleation process promoted by lipid membranes (43). These oligomers can then either redissolve or mature and elongate into highly structured amyloid fibrils (44). Once fibrils have been formed, secondary processes, including fragmentation and secondary nucleation, accelerate the aggregation process (45). Downstream to fibril formation, the mechanism of Lewy body formation and maturation remains to be established, although it is likely to involve interaction of different α-synuclein species with lipid membranes (21, 22, 46). The post-translational modifications of α-synuclein can affect essentially all the steps in the aggregation process, including by modulating the binding of α-synuclein to lipid membranes, which may increase the local concentration of α-synuclein and facilitate the initial nucleation events, compared to the slow nucleation rate of free monomers in solution (47).

α-Synuclein is an amyloidogenic protein that can forms characteristic cross-β amyloid fibrils (42). The aggregation process of α-synuclein can be followed *in vitro* as a function of time by measuring the fluorescence of amyloid-binding dyes, and appears a sigmoidal curve with lag, exponential and plateau phases. This macroscopic behaviour is the result of a complex interplay between different microscopic processes, which include primary nucleation, which is usually promoted by the presence of lipid membranes, fibril elongation, and secondary nucleation whereby the surfaces of existing fibrils catalyse the formation of new seeds (Figure 2) (45, 48). Since aggregates of α-synuclein are toxic and enhance the formation of further aggregates, blocking this process is a promising therapeutic route (39). Amyloid aggregation is toxic in multiple ways, including in particular through the formation of misfolded oligomers, which can disrupt mitochondrial and synaptic functions, and induce endoplasmic reticulum (ER) stress and membrane damage (49–52). In addition, larger α-synuclein deposits can sequester key cellular proteins and with consequential loss of function (21, 22, 53), and travel from cell to cell (54).

α-Synuclein does not readily aggregate spontaneously (55, 56) and this process is affected by the environmental conditions (57–59). The binding to lipid membranes offers a possible interface for primary nucleation events to initiate the toxic cascade of α-synuclein aggregation (47, 55). For example, aggregation has been shown to be accelerated by exosomes (60–62).

### Intersection of α-Synuclein Homeostasis and Lipid Homeostasis

Although Parkinson's disease is often described a proteinopathy, emerging evidence suggests that it could be considered as a lipidopathy, or most likely as a combination of the two (63). Multiple links between lipid homeostasis and genes associated with Parkinson's disease have been reported (64). An analysis of three genome-wide association studies (GWAS) revealed four major processes relevant to Parkinson's disease—oxidative stress response, endosomal-lysosomal functions, ER stress response, and immune response, with lipids and lipoproteins being key to all four processes (65). A link has also been uncovered between Parkinson's disease and *PLA2G6* (*PARK14*), the gene encoding phospholipase A2 group VI (PLA2G6). This enzyme catalyses glycerophospholipid and phospholipid hydrolysis to produce free fatty acid and lysophospholipids (66). Mutations in the *GBA* gene, encoding β-glucocerebrosidase (GBA), are a common risk factor for Parkinson's disease for both homozygous and heterozygous carriers. GBA catalyses the breakdown of glucocerebroside into glucose and ceramide, and a loss of function leads to the accumulation of glycosphingolipids (67, 68), influencing the sensitivity of neurons to α-synuclein aggregation and spreading (69).

**Figure 3:**
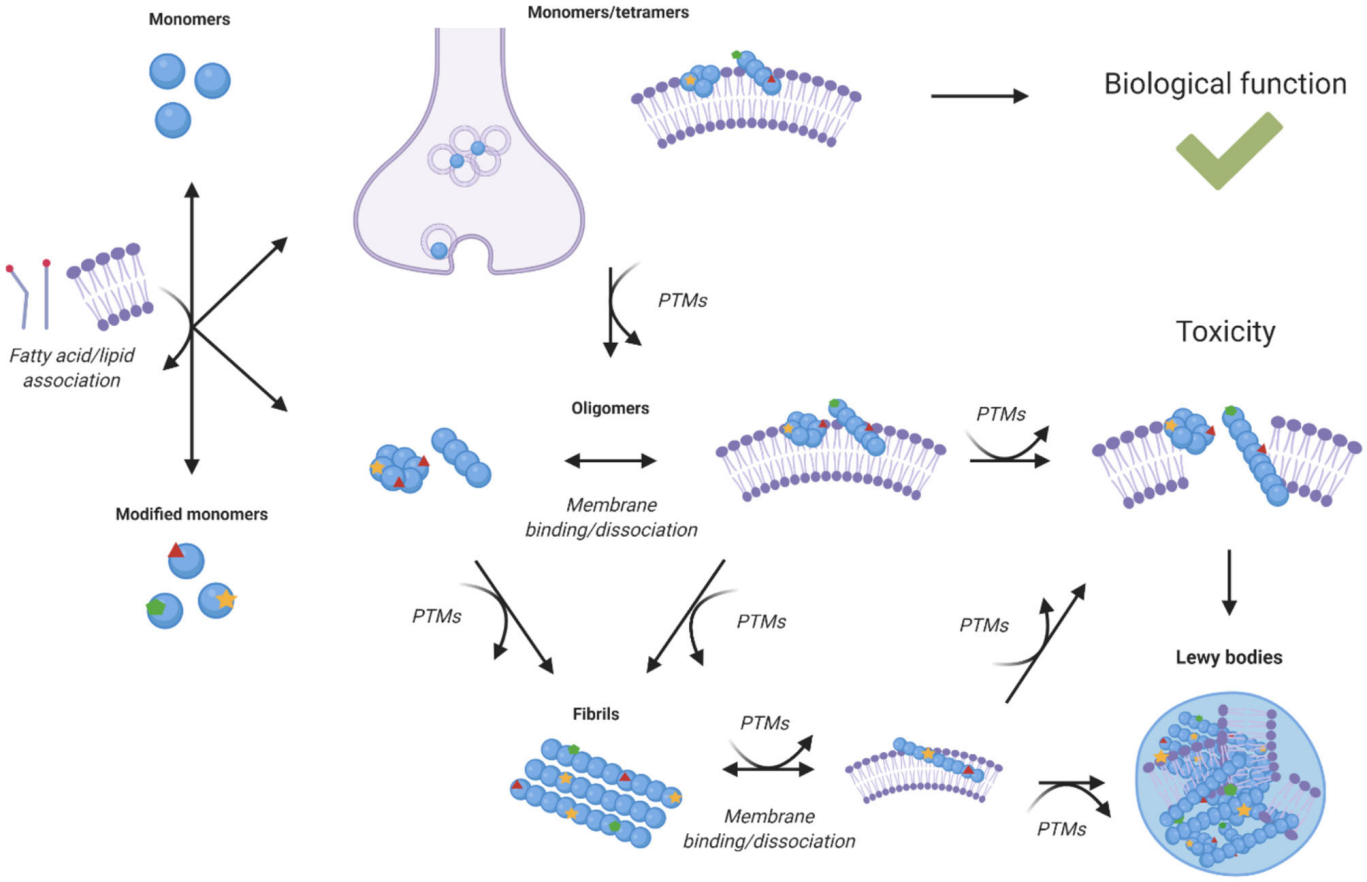
Effects of post-translational modifications on the relationship between α-synuclein homeostasis and lipid homeostasis. Lipid binding is associated with the cellular functions of α-synuclein, but it is also with its toxicity upon aggregation. Different species of α-synuclein can interact with cell membranes, lipid vesicles, and free fatty acids. The interactions of aggregated species of α-synuclein with lipid membranes can lead to altered structure, composition, function, and integrity of the membranes themselves (73–75, 77, 84–88, 90, 91). The binding of α-synuclein to lipids and lipid membranes can also impact its aggregation, toxicity, expression, and localisation. Post-translational modifications can alter the interaction of α-synuclein species with lipid species, modulate the binding, and dissociation of α-synuclein with lipid membranes and influence the aggregation propensity of α-synuclein (15, 16, 47, 55, 80–83, 92, 94).

In knock-out mice models, loss of α-synuclein function is not particularly detrimental, but *SCNA* triplication or α-synuclein mutations that affect its expression have been linked with Parkinson's disease (8). Although a gain of toxic function by α-synuclein was initially thought to involve primarily the processes of amyloid aggregation, recent studies have shown an increasing role for lipid homeostasis in α-synuclein induced toxicity (Figure 3). In this perspective, all the known familial mutations of α-synuclein occur within its lipid membrane binding region. A study in yeast found that α-synuclein toxicity was not dependent on fibril formation, and that mutations that lead to defective lipid membrane binding were less toxic, suggesting toxicity was caused by interaction with cell membranes (71). Another study observed α-synuclein toxicity correlated with accumulation of lipid vesicles in yeast, which induced ER to Golgi trafficking defects, suggesting that the reliance of neurons on lipid vesicle transport may be the reason they are so vulnerable to α-synuclein toxicity (72).

The existence of a soluble conformation of α-synuclein with α-helical secondary structure was recently reported and suggested to originate from transient interaction with lipid vesicles and may be the species that initiates aggregation (73). In the familial mutant E46K, one of the KTKEGV motifs important for lipid membrane binding is disrupted, and so this mutation has been utilised to study the effects of α-synuclein membrane binding. Two further mutations of E to K within other KTKEGV motifs have been made in E46K α-synuclein to create a binding-deficient α-synuclein form termed 3K (74, 75). In neurons, 3K α-synuclein led to increased inclusion formation (74) and in mice to a Parkinson's disease-like phenotype with inclusions containing α-synuclein and lipid vesicles (75).

Recently, it was observed that α-synuclein overexpression in yeast, rodent neurons and induced pluripotent stem cells (IPSCs) led to changes in cellular lipid profiles by increasing the formation of mono-unsaturated fatty acids (MUFAs). Surplus MUFAs, specifically oleic acid, subsequently induced enhanced α-synuclein toxicity by altering the equilibrium of membrane bound to soluble α-synuclein. These findings revealed a new therapeutic target for ameliorating Parkinson's disease, as stearoyl-CoA-desaturase inhibition could reduce biosynthesis of MUFAs and lead to reduced α-synuclein cytotoxicity (63, 76). α-Synuclein oligomerisation was found to be regulated by poly-unsaturated FA (PUFA) levels in neural cells, and deletion of the *SNCA* gene in mice led to a decrease in docosahexaenoic acid (DHA) and α-linolenic acid (ALA) (77). Free fatty acids, specifically DHA and ALA, have been shown to bind to α-synuclein and increase its aggregation at low ratios, but to reduce it at high ratios. α-Synuclein binding to DHA and ALA was protective against oxidative damage induced by fatty acids (15, 78–80). Altered levels of PE, PI, PS, and PC have been observed in Parkinson's disease patients (63). Under the oxidative stress conditions seen in Parkinson's disease, the balance between the cholesterol derivatives 24-hydroxysterol and 27-hydroxysterol can become disrupted (15, 81–83). These results suggest that the levels, modifications and toxicity of α-synuclein are intricately linked with lipids *in vivo*.

α-Synuclein has been shown to bind to mitochondrial membranes and stimulate mitochondrial tubulation, fragmentation and dysfunction (84–86). Mitochondrial dysfunction caused by α-synuclein leads to an increase in reactive oxygen species which may in turn lead to increased toxicity of α-synuclein (86). More generally, α-synuclein can remodel lipid membranes, for example POPG vesicles were remodelled into tubules (87), POPC/POPA bilayers were thinned (88), POPG vesicles remodelled into nanoparticles (89), sphingomyelin was remodelled into nanodiscs (90), and arachidonic acid was released from liposomes (91), upon interaction with α-synuclein.

### Modulation of the Interactions Between α-Synuclein and Lipid Membranes

The interaction of α-synuclein with lipid membranes is modulated by the properties of the phospholipid head groups. For example, 27-hydroxycholesterol, a product of cholesterol oxidation, induces expression and accumulation of α-synuclein in human dopaminergic neurons via transcription (82), dioleoyl-PA induces aggregation of α-synuclein (92), docosahexaenoic acid (DHA) can induce oligomerisation (80), and cholesterol modulates the binding of α-synuclein to vesicles (16). The lipid composition of cell membranes can affect the way in which α-synuclein interacts with the membranes themselves, as demonstrated by the findings that cardiolipin can promote the pore-forming activity of α-synuclein oligomers (93). The familial mutants of α-synuclein have been shown to have different membrane interaction properties (94), so small chemical changes can have an impact on how α-synuclein binds and responds to lipid membranes.

## Post-Translational Modifications of α-Synuclein

**Figure 4:**
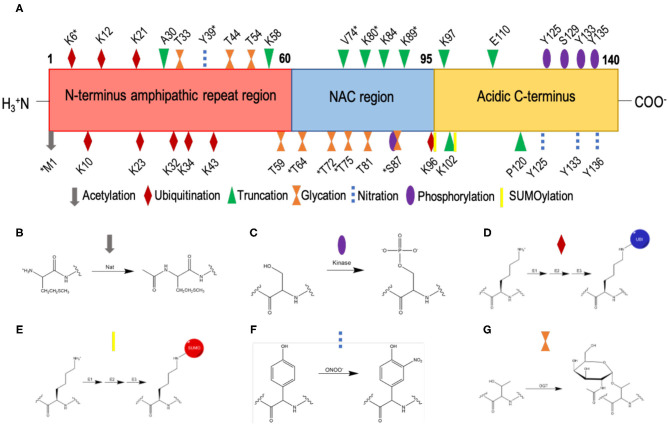
List of currently known post-translational modifications of α-synuclein. **(A)** The amino acid sequence of α-synuclein comprises an N-terminal amphipathic region (residues 1-60), a non-Aβ component (NAC) region (residues 61-95), and an acidic C-terminal region (residue 96-140). Post-translation modification sites are labelled; *indicates post-translation modifications within low-solubility areas using CamSol (70). **(B)** N-terminal acetylation, which is carried out by N-acetyltransferases (Nat). **(C)** Phosphorylation, which occurs by the esterification by a phosphate group of the side-chain hydroxyl moiety of serine or threonine residues; shown here is the phosphorylation of a serine residue. **(D)** Ubiquitination, where ubiquitin is added to lysine residues via an isopeptide bond, catalysed by sequential action of 3 enzymes, E1 (ubiquitin-activating enzyme), E2 (ubiquitin-conjugating enzyme) and E3 (ubiquitin ligase). **(E)** SUMOylation, where SUMO is added to lysine residues via a thioester bond, catalysed by sequential action of 3 enzymes, E1 (SUMO-activating enzyme), E2 (SUMO-conjugating enzyme), and E3 (SUMO ligase). **(F)** Nitration, where tyrosine residues are nitrated by peroxynitrite; the phenolic R group is converted to 3-nitro-tyrosine, by the addition of a nitro (NO_2_) group onto the ortho position of the ring by peroxynitrite. **(G)** O-GlcNAcylation, where the enzyme O-GlcNAc transferase (OGT) catalyses the addition of N-acetylglucosamine (GlcNAc) to the hydroxyl group (O-linked) of the side-chains of serine and threonine residues; shown here is the O-GlcNAcylation of a threonine residue.

α-Synuclein is subject to multiple post-translational modifications (Figure 4) including acetylation, phosphorylation, ubiquitination, SUMOylation, nitration, truncation, and glycation (49, 95–97). Much work has been undertaken to understand how these post-translational modifications affect the aggregation of α-synuclein (98). By contrast, the impact of post-translational modifications on the affinity of α-synuclein for biological membranes requires further study.

### N-Terminal Acetylation

N-terminal acetylation is a common co-translational and post-translational modification, which affects about 70–90% of the proteins in eukaryote proteomes (99, 100). In humans, there are 7 types of N-terminal acetyltransferases (NatA-F and NatH), which are enzymes that catalyse the covalent attachment of an acetyl group (CH_3_CO) to the free α-amino group (${\text{NH}}_{3}^{+}$) of the N-terminal residue of a protein, depending on its N-terminal dipeptide (101). This post-translational modification impacts a wide range of properties of proteins, including their stability, folding, interactions and subcellular localisation, and has been implicated in a wide range of human disorders, including cancer and developmental diseases (102).

It has been estimated that over 80% of α-synuclein molecules are constitutively N-terminal acetylated (103), the large majority of which by the N-acetyltransferase B (NatB) complex (104) (Figures 4A,B). The addition of the acetyl group to the amine group of the N-terminus results in the loss of a positive charge in an area of poor solubility (105). Given that phospholipid headgroups tend to be negatively charged, modifications in this region impact the binding behaviour of α-synuclein to cell membranes. In a recent mass spectrometric analysis, Lewy bodies have been found to be enriched in N-terminal acetylated α-synuclein (106).

In addition, as N-terminal acetylation induces more α-helical structure in monomeric α-synuclein in solution (107), the entropic barrier from intrinsically disordered free α-synuclein to a membrane bound α-helical α-synuclein may be lowered. Indeed, N-terminal acetylation has been shown to generally enhance the affinity of α-synuclein for lipid membrane binding (Table 1). Recently, it was shown by nuclear magnetic resonance (NMR) spectroscopy that N-terminal acetylation increases the binding affinity but does not alter not structure of the bound form α-synuclein to synaptic vesicle mimics (DOPE:DOPS:DOPC SUVs, 5:3:2 w/w) (108). Similarly, an enhanced binding affinity to synaptic-like vesicles has been reported, when α-synuclein is N-terminally acetylated (111, 112). N-terminal acetylation was observed to not only effect the interaction of the N-terminus of α-synuclein with phospholipid vesicles, but also to impact the entire protein (112). However, no significant difference was observed in the membrane binding affinity in acetylated and non-acetylated α-synuclein with synaptic vesicle mimics (POPC/POPS/Chol large unilamellar vesicles, LUVs) (113).

**Table 1:** Summary of the known post-translational modifications of α-synuclein, and of their effects on its binding to lipid membranes.

**Post-translational modifications**	**Position**	**Lipid membranes**	**Reported effects on lipid membranes**	**Reported functional impact**	**References**
Acetylation	M1	DOPE/DOPS/DOPC synaptic-like vesicles	Increases the affinity but does not affect the structure	Regulates the binding to synaptic vesicles	(108)
		High-curvature synaptic-like vesicle	“	“	(109)
		SUVs with lipid rafts (DOPC/SM/Chol)	“	“	
		Giant cell membrane-derived plasma vesicles	“	“	(110)
		DOPE/DOPS/DOPC synaptic-like vesicles	“	“	(111)
		Non-ionic detergent BOG	“	“	(112)
		DOPE/DOPS/DOPC synaptic-like vesicles	“	“	
		Synaptosomal membranes	No difference		(113)
		POPG LUVs	“		
		POPC/POPS/Chol LUVs synaptic-like vesicles	“		
		HeLa cell cellular membranes	“		
Phosphorylation	S129	Synaptosomes from mouse brains	Does not affect the wild-type, but affects A30P (increase) and A53T (decrease) membrane binding		(114)
		SN dopaminergic neurons internalised vesicles	Disruption of vesicles increased by fibrils with pS129	Upregulation of the pathology	
	Y39	SUV synaptic vesicle mimic	Disruption of the helix-2 binding region	May lead to interaction with other proteins and vesicles, resulting in aggregation	(115)
	S87	POPG vesicles and SDS micelles	Decreases binding affinity	“	(116)
	S129	POPG vesicles and SDS micelles	No difference		
	S129	POPG	Fibril-induced rupture of lipid membranes	Upregulation of pathology	(117)
Ubiquitination	K63	Endosomal membranes	Increases internalization and localisation	Regulation of degradation	(118)
	K6	POPG vesicles	No difference		(119)
SUMOylation	Unspecific	Extracellular vesicles	Enhances binding	May enhance spreading	(120)
Nitration	Y39	POPG	Decreases binding affinity	May increase interactions with other proteins and lipid vesicles, leading to aggregation	(121)
	Y125				
	Non-specific	POPC/POPA UVs	“	“	(122)
	Y39	POPC/POPS 1:1	“	“	(123)
Truncation	1-119	DMPS SUVs	Aggregates an order magnitude faster with SUVs	Increased aggregation	(124)
	1-103	DMPS SUVs	Aggregates form mature fibrils rather than kinetically trapped protofibrils	Possible competent seed for secondary nucleation *in vivo*	
	1-121	POPG vesicles erythrocytes	Decreases the ability to disturb lipid membranes	Protective in pathogenesis	(125)
Glycation	S87	POPG	No effect on binding affinity		(126)
	S87	POPS	“		
	T72	POPG	“		(127)
	T72	POPS	“		
	T75	POPG	“		(128)
	T81	POPG	“		
	S87	POPG	“		
	T72	POPG	“		
	T72/75/81	POPG	Slight decrease in affinity	Possible regulation of membrane binding	

A study of LUVs comprised of POPG and mouse synaptosomes found no significant effects of N-terminal acetylation on α-synuclein membrane binding (113). Binding to SDS micelles was also examined, but again no significant effect was detected, although this modification induced a new conformation of α-synuclein when bound to β-octyl-glucoside (BOG) vesicles (111). It was also found that acetylation of α-synuclein enhances binding to phospholipid vesicles that have similar size and high curvature to synaptic vesicles (15:0, 12:0 lyso-, and 16:0 lyso-PC SUVs). A decrease in binding affinity to DPPC vesicles containing cholesterol was also observed when α-synuclein is acetylated, and enhanced binding to lipid raft SUVs with high curvature (DOPC/SM/Chol) (109). An investigation of the effects of N-terminal acetylation on α-synuclein in yeast revealed that N-terminal acetylation was required for proper plasma membrane targeting (129). N-terminal acetylation has been shown to increase the binding affinity of N-terminus of α-synuclein to giant cell membrane-derived plasma vesicles (110).

### Phosphorylation

Phosphorylation is the most common post-translational modification of eukaryotic proteins, which acts as a molecular switch to regulate protein interactions (130, 131). This process is carried out by kinases, which are a large class of enzymes (132) and reversed by phosphatases (133). The phosphorylation of proteins is an esterification reaction that involves the attachment of a phosphoryl group to the hydroxyl group of the side-chains of specific amino acids, most commonly serine, threonine and tyrosine, and in some cases arginine, lysine, aspartic acid, glutamic acid and cysteine (130, 134). The dysregulation of protein phosphorylation is associated with a wide range of human diseases, and kinases are a major target for pharmacological intervention, in particular for cancer (135, 136).

α-Synuclein can be most commonly phosphorylated at serine and tyrosine residues (Figures 4A,C) and it is typically phosphorylated at S129 and S87 in Lewy bodies (103). The impact of phosphorylation can be studied by co-expressing kinases to phosphorylate α-synuclein, or by using phosphomimetics such as mutations to aspartic acid or glutamic acid. Alternatively, since kinases may lead to unspecific phosphorylation, semi-synthetic strategies (137), or post-translational chemical mutagenesis (138) can enable site-specific phosphorylation.

Phosphorylation at S129 (pS129) is closely linked to Parkinson's disease (139–141) and increases to about 90% levels in Lewy bodies from the 5% levels observed in healthy brains (142). However, it still remains to be established whether phosphorylation at S129 precedes Lewy body formation or follows it as a downstream event, whether it inhibits or promotes α-synuclein aggregation, and whether it enhances or reduces α-synuclein binding to lipid membranes. *In vitro* studies have offered conflicting conclusions about the effect of pS129 on α-synuclein aggregation (114, 143). By co-expressing polo-like kinase 2 (PLK2) with α-synuclein to phosphorylate S129, an increased rate pS129-α-synuclein aggregation was observed, but synaptosome membrane binding was not affected (114). However, the mutants A30P and A53T were affected, as A30P-α-synuclein binding increased and A53T-α-synuclein binding was lowered by the presence of pS129. The internalisation of pS129-α-synuclein fibrils was not significantly different with respect to unmodified α-synuclein in dopaminergic neurons in the ventral tegmental area but was increased in those in the *substantia nigra*. Disruption of lipid membranes was also increased by S129 phosphorylation of A30P-α-synuclein (114). By synthetic phosphorylation at S129 it was found that this modification induced a different strain and increased the toxicity of fibrils, as pS129-α-synuclein fibrils more readily ruptured POPG vesicles (117). Monomeric pS129-α-synuclein bound to POPG vesicles exhibited less α-helical secondary structure, suggesting that phosphorylation may impede lipid membrane binding (117). Aggregates of pS129-α-synuclein were found to co-localise with mitochondria, impairing their function (144).

A study of the influence of phosphorylation of Y39 on synaptic vesicle mimics (DOPS/DOPC/DOPE SUVs) revealed that pY39 disrupted binding but had no effect when binding to SDS micelles. It was also found the phosphomimetic Y39E perturbed peripheral lipid membrane localisation compared to wild-type α-synuclein in yeast. The effects of pY39 were similar to that of the familial mutation G51D (115). An analysis of the effects of phosphorylation at S87 showed that pS87 enhanced conformational flexibility, reduced aggregation, and decreased α-synuclein binding to lipid membranes (SDS micelles and POPG vesicles) (116).

Phosphorylation at different residues may impact in distinct manners the interaction of N-terminus of α-synuclein with phospholipid membranes. Since residues S87 and Y39 lie within the α-helical domain when α-synuclein binds to membranes (5, 11) and in an areas of low solubility (Figure 4A), they may have more of an effect on the binding affinity of the N-terminus of α-synuclein and the resulting aggregation of the protein. It is still unclear when phosphorylation occurs in the aggregation process, but it has been suggested to happen during or post aggregation, rather than before (116, 145–147). The disruption of phosphorylation is linked with Parkinson's disease, with the mutation of PARK6 encoding an protein kinase PINK1 being the second most common cause of autosomal recessive familial Parkinson's disease (148).

### Ubiquitination

Ubiquitination is a reversible post-translational modification that is essential for the maintenance of protein homeostasis. Ubiquitin is a 76-residue protein that is added via an isopeptide bond to lysine residues to its target as a marker for degradation or to alter the targets interactions (149) (Figure 4D). The process is mediated by three enzymes E1 (ubiquitin-activating enzyme), E2 (ubiquitin-conjugating enzyme), and E3 (ubiquitin ligase) and ubiquitin can be removed by deubiquitinating enzymes (150). Poly-ubiquitin chains can be built up on a protein via sequential addition of ubiquitin to one of the seven lysine residues within the previous ubiquitin molecule (151). The specificity of the ubiquitin-lysine linked chains influences the effect that ubiquitination has on the target, for example K48-linked ubiquitin chains trigger degradation of the target, whereas K63-linked ubiquitin chains regulate the formation of complexes (149). The ubiquitination system is known to be deregulated in diseases such as cancer, immune disorders, muscle-wasting disorders, diabetes, and neurodegeneration (152).

Lewy bodies have shown immunoreactivity to anti-ubiquitin antibodies and contain ubiquitin-α-synuclein (103, 153). α-Synuclein has eight lysine residues that can be ubiquitinated (Figure 4A), and ubiquitin has multiple internal lysine residues from which poly-ubiquitin chains can form. The combinations of ubiquitin-α-synuclein sites and linkages are vast, and so there is much more to learn about the effects of different ubiquitination on α-synuclein, especially on the interactions of N-terminus of α-synuclein with phospholipid membranes.

Multiple groups have found that ubiquitination of α-synuclein affects the aggregation. Tetra- ubiquitinated K-48 linked chains at K12 of α-synuclein was shown to increase aggregation propensity of α-synuclein, but the aggregates formed were amorphous, and did not convert into mature fibrils (154). The effects on mono-ubiquintation at multiple lysine residues in α-synuclein were investigated by mutating K to C to allow the addition of ubiquitin via a disulphide-mediated reaction (155). This study revealed that the ubiquitinated lysine residue was capable of modulating the aggregation of α-synuclein. K10C-ubiquitin and K23C-ubiquitin reduced the rate of α-synuclein fibril formation, and that K6C-ubiquitin, K12C-ubiquitin and K21C-ubiquitin had a moderate inhibitory effect and K32C-ubiquitin, K34C-ubiquitin, K43C-ubiquitin, and K96C-ubiquitin had strong inhibitory effects (155).

In a recent study, mono-ubiquitin was synthetically added to α-synuclein via a BTA linkage, to allow ubiquitination to remain in reducing environments (156). This approach revealed that ubiquitination at K6, K23, K43, and K96 had no effect on monomeric α-synuclein change to secondary structure via CD, but all reduced amyloid aggregation. Furthermore, the ubiquitin-α-synuclein aggregates were less toxic to SH-SY5Y cells (156). In a related study, lysine residues of α-synuclein were semi-synthetically modified via disulphide directed mono-ubiquitination. This approach showed that K6-ubiquitin and K23-ubiquitin both inhibit fibril formation, but do not alter the structure of fibrils. However, K96-ubiquitin inhibits aggregation and also alters structure of aggregates formed (157).

The effect of ubiquitin on the interaction of α-synuclein with phospholipid membranes revealed the mono-ubiquitination at position K6 along the sequence of α-synuclein inhibited aggregation of monomeric α-synuclein, but did not affect the secondary structure when bound to POPG vesicles (119). However, it was also found that K63 poly-ubiquitinated α-synuclein was preferentially internalised and translocated to endosomes (118). It is worth noting that lysine residues within the KTKEGV regions are thought to be essential in the mechanism of α-synuclein binding to anionic membranes, and so ubiquitination of these residues may prevent membrane binding of monomers, oligomers, and fibrils (158).

### SUMOylation

Related to ubiquitination, the addition of small ubiquitin-like modifier (SUMO) proteins is a reversible, covalent, post-translational modification of lysine residues (159). SUMOylation acts to regulate the function, localization, and interactions of the modified proteins. There are several SUMO proteins within the SUMO family, which is highly conserved across eukaryotes, with the most abundant SUMOylation found in mammalian cells being SUMO-1 addition (160). Similarly to ubiquitination, SUMOylation is carried out by the sequential action of three enzymes, E1, E2, and E3, while de-SUMOylation is catalysed by SUMO-specific proteases (150, 160). This post-translational modification is carried out through the conjugation of the ε-amino group of a lysine residue within the target protein via a thioester bond to a C-terminal glycine in a SUMO protein (Figure 4E) (161). SUMOylation of lysine residues occurs at consensus sequences consisting of Ψ-K-x-D/E, where Ψ is a branched aliphatic amino acid, x is any amino acid, and K is the modified lysine (162). Protein SUMOylation has been linked to a variety of diseases including cancer, heart disease, and neurodegeneration (160, 163, 164).

SUMOylation of α-synuclein is closely linked to Parkinson's disease (165). SUMO proteins have been detected in Lewy bodies as colocalised with α-synuclein (166). In α-synuclein there are two major sites for SUMOylation (Figure 4A), K96 and K102 (167), although seven more lysine residues have been reported to be SUMOylated (168). It was also shown that α-synuclein aggregation was stimulated by SUMOylation upon proteasomal inhibition. SUMO proteins have been observed to co-localise with lysosomes and may recruit them to aggregated α-synuclein (169), or target α-synuclein to the autophagy pathway (170). α-Synuclein mixed with SUMO-α-synuclein was found to be less prone to aggregation compared to wild-type α-synuclein, even if a small fraction of α-synuclein monomers had been SUMOylated, leading to the suggestion that a role of SUMOylation is to increase the solubility of α-synuclein (168). Similarly, synthetically SUMOylated α-synuclein at K96 and K102 was observed to reduce amyloid aggregation in a site-specific manner (156, 171). The amorphous aggregates of K102-SUMO α-synuclein were also found to be significantly less toxic to SH-SY5Y cells than the amyloid aggregates of wild-type α-synuclein (156).

The observation that the release of α-synuclein within extracellular vesicles is SUMO-dependent led to the suggestion that SUMOylation acts as a regulator of α-synuclein sorting into the extracellular vesicle pathway which may facilitate the spreading of α-synuclein pathology (120). The affinity of SUMO for SUVs was shown to vary depending on the phospholipid makeup, with SUMO having a high affinity for the phospholipids PI (3)P, PI (3–5)P3, and POPS, but a lower one for uncharged POPC. SUMOylated-α-synuclein was also found to be enriched in the mouse neuroblastoma N2A cell membrane pellets compared to that of unmodified α-synuclein, suggesting that SUMOylation promotes membrane binding (120). Three familial variants of α-synuclein (A30P, A53T, and E46K) were found to be more susceptible to PIAS2 (an E3 ligase) mediated SUMOylation *in vitro* and in SH-SY5Y cells, and this was associated with an increase in aggregation and inclusion formation. It was also observed that SUMOylation of α-synuclein decreased the ubiquitination both *in vitro* and *in vivo* (172).

SUMOylation is an essential, intricate and dynamic process, the complexities of which are still not fully understood. Although little is known about how SUMOylation affects the binding and function of α-synuclein at phospholipid membranes, it is clear SUMOylation has an impact on α-synuclein aggregation. To fully understand the role of SUMOylation in Parkinson's disease, it will be important to investigate how the membrane binding of α-synuclein is affected as this is a possible site for aggregation and key to the function of α-synuclein.

### Nitration

Nitration is an irreversible post-translational modification that occurs on tyrosine residues, in particular in the presence of nitric oxide radicals (173). In this process, the phenol group of tyrosine is converted to 3-nitro-tyrosine in a reaction mediated by reactive nitrogen species, such as peroxynitrite (ONOO^−^, Figure 4F), an oxidant formed by the reaction of the nitric oxide and superoxide radicals (^.^NO and ${\text{O}}_{2}^{-}$) (174). These radicals are normally rapidly removed by superoxide dismutases (SODs), but can form spontaneously peroxynitrite via a diffusion-limited reaction (175, 176). Nitration is often highly selective and depends on the structure and environment surrounding the tyrosine residues (177). This post-translational modification has been associated with over 50 diseases, including cancer, cardiovascular disorders, and neurodegeneration (178) and can affect proteins structure, function, and other post-translational modifications (179).

α-Synuclein has four tyrosine residues (Y39, Y125, Y133, and Y136), which can be nitrated to form nitrated-α-synuclein (Figure 4A) (180). Nitrated α-synuclein is enriched in Lewy bodies compared to control brains (181, 182). Nitrated tyrosine residues are reactive and can form dityrosine bonds via cross-linking, which can induce oligomerisation of α-synuclein (183). Nitrated α-synuclein has been shown to induce cytotoxicity in SH-SY5Y cells (184), and in the *substantia nigra* of rats (185). It was also found that Y39 is preferentially nitrated (nY39) in a cell model of Parkinson's disease overexpressing monoamine oxidase B (MOA-B), an enzyme involved in dopamine metabolism and known to create reactive oxygen species, and that this effect can be abrogated using selegiline, an inhibitor of MOA-B approved for the treatment of Parkinson's disease (186). Furthermore, using the Y39F mutant, it was shown that nY39 is key to dityrosine cross-linking and the subsequent induction of oligomer formation in α-synuclein (121). Therefore, assessing the effects of nitration could help develop a better understanding of the pathogenesis of Parkinson's disease.

To study the effects of nitration on α-synuclein one can expose α-synuclein to nitrating substances such as peroxynitrite or tetranitromethane (122, 187, 188), leading to unspecific nitration. A study of the effects of non-specific nitration revealed that in the presence of phospholipid vesicles (POPC/POPA SUVs), nitrated-α-synuclein bound with lower affinity than wild-type α-synuclein. It was also observed that nitration induced a change in secondary structure, an increase in disorder in solution, and a decreased propensity to adopt α-helical structures in presence of phospholipid vesicles. The effects of non-specific nitration were found to be largely due to Y39, as the mutation Y39F ameliorated them (122).

As an alternative non-specific nitration, one can use semi-synthetic or mutational approaches to individually modify one tyrosine residue to examine site-specific effects (121, 123). The selective nitration at Y39 was obtained by mutating the other three tyrosine residues in α-synuclein to phenylalanine residues (123). It was thus demonstrated that nY39 decreased the binding affinity of α-synuclein to negatively charged lipid vesicles, due to the electrostatic repulsion of the partial negative charge of the nitro group. With the mutant Y39F, it was also shown that although the C-terminus is not directly involved in binding phospholipid vesicles, but that nitration of Y125, Y133, and Y136 disrupted the binding affinity of α-synuclein. The disruption of binding by C-terminal nitration was attributed to nitration leading to a compaction of the C-terminus that may disrupt the long range contacts and allosterically regulates binding of α-synuclein to phospholipids (123). Further, by nitrating residues Y39 or Y125, a significant reduction in the α-helix formation upon α-synuclein binding to POPG vesicles was observed, while nitration at Y39 or Y125 lead to the formation of fibrils with distinct morphology compared to that of wild-type α-synuclein. It was also found binding to liposome-containing phospholipids protected α-synuclein against nitration (189).

### Proteolytic Cleavage and C-Terminal Truncations

Protein truncations can result from a variety of causes, including genetic variations, post-translational modifications, or incomplete degradation. Truncations can alter the structure and interactions of proteins, and cause loss or gain of function (190, 191). Abnormal proteolytic processing is a common feature of proteins that drive neurodegenerative diseases, which may be induced by the release of proteases from their cellular compartments under conditions of stress (192). Notably, the aberrant proteolytic processing of amyloid precursor protein (APP) is linked to Alzheimer's disease by producing an excess of the aggregation prone 42-residue form of the amyloid-β peptide (193). Protein truncations are also prevalent in Pick's disease, Huntington's disease, spinocerebellar ataxia, amyotrophic lateral sclerosis and Parkinson's disease (192).

Irreversible truncation of α-synuclein at the N and C termini is common, with over 15% of α-synuclein in Lewy bodies being truncated (106, 194–196). Truncation is thought to occur due to incomplete digestion of α-synuclein by a number of enzymes, including calpain, plasmin, neurosin, 20S proteasome, cathepsin D, caspase 1, matrix metallo proteinase-1, and asparagine endopeptidase (Figure 4A), and may be promoted by dysfunction of protein homeostasis machinery such as the lysosomal chaperone-mediated autophagy (194, 197–205). The N-terminal of α-synuclein is essential for binding to phospholipid membranes, and so truncation at N-terminal sites may reduce or inhibit cell membrane binding. Truncation can stimulate aggregation and toxicity *in vitro* (198, 206–208) and *in vivo* (197, 209–211) and increase prion-like spreading (197, 208).

The impact of C-terminal truncations (α-synuclein 1-119 and 1-103) on the aggregation of α-synuclein in the presences of phospholipid vesicles was recently investigated (124). Similar secondary structures were observed for both truncated variants and wild-type α-synuclein. α-Synuclein 1-119 aggregated in a comparable way, but an order of magnitude faster than the wild-type form. However, the α-synuclein 1-103 variant aggregated following a distinct mechanism forming morphologically different aggregates that resembled mature fibrils compared to the protofibrils produced by the aggregation of wild-type α-synuclein in the presence of DMPS SUVs (124). An analysis of the biophysical properties of α-synuclein 1-121 revealed that in the proximity of POPG vesicles both the secondary structure and aggregation of full-length or truncated α-synuclein were similar. With both POPG vesicles and erythrocytes, truncated α-synuclein exhibited a decreased ability to distort the phospholipid membranes. Conversely, α-synuclein 1-121 was found to have higher toxicity compared to full-length α-synuclein, possibly following the activation of apoptosis signalling pathways and upregulation of phosphorylation at S129 of α-synuclein (125).

Truncation can occur at multiple sites along the sequence of α-synuclein (Figure 4A) (196) but the effects many of these modifications are not known in detail. In all cases, C-terminal truncation of α-synuclein reduces the solubility of α-synuclein in solution and affects its membrane binding properties. Further research, however, will be required to fully understand how truncations affect the behaviour of α-synuclein in the cellular milieu. A distinct species of α-synuclein, termed p-asyn^*^, truncated at both N- and C-termini and phosphorylated, was detected in Lewy bodies in mice models of Parkinson's disease and primary neurons exposed to α-synuclein fibrils (212). It was found that p-asyn^*^ preferentially associated with mitochondria and ER to induce toxicity (212).

### O-GlcNAcylation

O-GlcNAcylation is a reversible enzymatic post-translational modification in which N-acetylglucosamine (GlcNAc), an amide derivative of glucose, is transferred from uridine diphosphate GlcNAc (UDP-GlcNAc) to the hydroxyl group of a serine or threonine side-chain in a protein (Figure 4G) (213). Thousands of proteins in the nucleus, cytoplasm and mitochondria have been identified to be targets of O-GlcNAcylation, which modulates their functions, interactions, and maintenance. The addition and removal of GlcNAc is catalysed by two enzymes, O-GlcNAc transferase (OGT) and O-GlcNAcase (OGA), respectively (214). The levels of O-GlcNAcylation depend on environmental stimuli, as well as on the levels of cellular glucose entering the hexosamine biosynthetic pathway to produce UDP-GlcNAc (215). O-GlcNAcylation is an important modification in the brain, and has been observed to modulate synaptic signalling, memory formation, and neuron growth (216) and is also key in cellular response to stress (217). The dysregulation of O-GlcNAcylation has been linked to metabolic diseases, cancer, cardiovascular diseases, and neurodegeneration (213).

At least nine residues of α-synuclein have been reported to be O-GlcNAcylated *in vivo* (128, 218), five of which are in the NAC region (Figure 4A). Semi-synthetic O-GlcNAcylation of α-synuclein at various positions inhibited aggregation, reduced toxicity of aggregates to both SH-SY5Y cells and rat primary cortical neurons (127). A study of the impact of S87 O-GlcNAcylation revealed a 5-fold reduction in fibril formation, and the formation of shorter fibrils (126). Single O-GlcNAcylation at T72, S87, T75 or T81, and of triple O-GlcNAcylation at T72, T75, and T81, reported site-specific effects (128). All mono-glycations had limited effects on the binding affinity of α-synuclein to lipid vesicles, whereas the triple-glycation decreased the helicity of α-synuclein upon membrane binding. All glycations inhibited seeded aggregation, with T81 and the triple-glycation having the most profound effects. Fibrils of both T75 and triple-glycations added to primary cultured mouse neurons with monomeric α-synuclein, were less toxic that wild-type α-synuclein (128). T72 and T75 are both within an area of low solubility in α-synuclein (Figure 4A) and so glycation at that site may increase the solubility of α-synuclein. These studies also showed that this modification has essentially no effect on phospholipid vesicle binding (126, 128). O-GlcNAcylation also impacted phosphorylation of α-synuclein, inhibiting S129 phosphorylation by CK1, PLK3, and GRK5 but increasing S87 phosphorylation by CK1 (127).

Overall, although O-GlcNAcylation does not affect α-synuclein binding to certain phospholipid membranes (128), other mixtures of phospholipids should be analysed, such as synaptic vesicle mimics, to determine if this effect would remain the same *in vivo*.

### Post-translational Modifications of Proteins Interacting With α-Synuclein

The behaviour of α-synuclein can also be affected by post-translational modifications of other proteins interacting with it. For example, palmitoylation, which is the post-translational addition of the fatty acid palmitate to cysteine residues, mediates the interactions of SNARE proteins with lipid membranes. Thus, although α-synuclein is not known to be palmitoylated, its interactions with lipid membranes can be affected indirectly by this post-translational modification (219).

## Post-Translational Modifications in the Diagnostics of Parkinson's Disease

Parkinson's disease has been traditionally diagnosed at the clinical level by the presence of motor symptoms, including bradykinesia, rigidity and resting tremor, and a range of non-motor symptoms, including constipation, anosmia, depression, and sleep disorder (220, 221). A definitive diagnosis is performed at the neuropathological level through the post-mortem detection of Lewy bodies (220). Without a well-established aetiology of the disease, however, its diagnosis remains challenging, as other movement disorders exhibit similar symptoms, such as multiple system atrophy and progressive supranuclear palsy (222, 223). Given this situation, it is crucial to establish accurate diagnostic methods at the molecular level, and indeed the search for biomarkers for Parkinson's disease has been a highly researched topic over the past several years (224).

Neuroimaging techniques offer a promising avenue of Parkinson's disease biomarker research (225), in particular through the use of positron emission tomography (PET) (226), although further developments are still required (227). In parallel efforts, many groups have focused on biomarkers present in biofluids, including cerebrospinal fluid (CSF), blood and saliva, or biopsies (228). The total levels of α-synuclein and of α-synuclein aggregate species have been investigated as potential biomarkers (229), and many more have been proposed, including through genetics, transcriptomics, and proteomics (227, 230, 231). In this review, we focus in particular on the opportunities offered by the detection and quantification of post-translationally modified forms of α-synuclein for the development of effective diagnostic tools (232).

### Phosphorylation

As phosphorylation of α-synuclein is highly prevalent in Lewy bodies, the extent of α-synuclein phosphorylation have been studied as a potential biomarker for Parkinson's disease. The levels of pS129-α-synuclein in CSF have been to be significantly increased in Parkinson's patients compared to controls (233–237). pS129-α-Synuclein levels in CSF have been correlated with disease progression as measured by clinical assessment through the Unified Parkinson's Disease Rating Scale (UPDRS) (233) and disease duration (238). pS129-α-Synuclein levels in blood plasma (236), olfactory mucosa (239, 240), salivary glands (241, 242) colonic biopsies (243), and skin biopsies (232, 244–246) have also been investigated.

### Nitration

Because of the links between α-synuclein nitration and Parkinson's disease, this modification has been investigated for the development of diagnostic tools for this condition. In a recent study, nitrated α-synuclein in salivary glands was detected in Parkinson's disease patients but not observed in healthy controls (247). Another study found the abundance of nitrated α-synuclein peripheral blood mononuclear cells to be significantly higher in Parkinson's disease patients than in controls. The levels of nitrated α-synuclein were also correlated with the those of reactive oxygen species, although no correlation was found between disease severity or duration (248). Nitrated α-synuclein levels were detected in colonic tissue and found to increase during ageing, and a loss of neurons was correlated with accumulation of both α-synuclein and nitrated α-synuclein (249).

### Other Post-translational Modifications

Further post-translational modifications of α-synuclein have received less attention, however some studies show promise in using post-translational modifications as biomarkers for Parkinson's disease. The abundance of post-translationally modified α-synuclein was studied in α-synuclein enriched erythrocytes extracts (250). Phosphorylated Y125, nitrated Y39, and lysine-glycated α-synuclein levels were found to be increased in Parkinson's disease, while SUMOylated α-synuclein levels were reduced. Combining all the post-translational modifications led to a predictive score of Parkinson's disease with increased sensitivity and specificity. Furthermore, each post-translational modification alone, and combined correlated with Parkinson's disease severity (UPDRS scores) and all but SUMOylation correlated with duration of disease (250).

The plasma levels of ubiquitin C-terminal hydrolase L1 (UCHL1) were significantly higher in late Parkinson's disease patients compared to healthy controls and the amount of UCHL1 correlated to disease severity. UCHL1 is associated with increased ubiquitin levels and stability in neurons (251). The abundance of truncated α-synuclein in platelets was also studied, but not found to be significantly different between Parkinson's disease patients and controls (252).

## Therapeutic Targeting of Post-Translational Modifications of α-Synuclein

Currently available treatments for Parkinson's disease are aimed at managing symptoms, but they do not stop the progression of the disease (253–256). Therapeutic interventions targeting α-synuclein aggregation and interactions offer promising opportunities for developing disease-modifying drugs (257). Since post-translational modifications modify how α-synuclein aggregates and interacts with lipid membranes, they offer promising opportunities for the treatment of Parkinson's disease and related synucleinopathies.

### Phosphorylation

The modulation of phosphorylation of α-synuclein is an important therapeutic target, in particular through the pharmacological modulation of kinases and phosphatases (258–260). Inhibition of the α-synuclein kinase c-Abl by nilotinib, an FDA-approved cancer treatment, enhanced clearance of α-synuclein in mice, protected neurons from α-synuclein toxicity and improved motor behaviour in a mouse model of Parkinson's disease (261). By increasing methylation of phosphoprotein phosphatase 2A (PP2A), the activity of PP2A was enhanced leading to decreased α-synuclein phosphorylation at S129, which decreased α-synuclein aggregation and toxicity in mice (262).

Leucine-rich repeat kinase 2 (LRRK2) has emerged from GWAS as one of the most important risk loci for Parkinson's disease (263, 264). LRRK2 is protein kinase that regulates secretory and endocytic vesicle trafficking by phosphorylating a group of RAB proteins (265). LRRK2 is associated with both the familial and sporadic forms of Parkinson's disease, with G2019S being the most common mutation, which increases its kinase activity (266). LRRK2 has been observed to co-localise with α-synuclein in nigral Lewy bodies (267, 268), and two studies reported an increase in phosphorylated α-synuclein inclusions in mice carrying the A53T-α-synuclein and G2019S-LRRK2 mutations (269, 270). Further studies in rodents detected higher levels of pS129-α-synuclein in the striatum in G2019S-LRRK2 compared to wild-type LRRK2 carriers (271, 272). However, other studies concluded that LRKK2 is unlikely to directly phosphorylate α-synuclein (263). Other protein kinases have also been linked to Parkinson's disease, including a cyclin G-associated kinase (GAK) encoded by the *GAK* gene, which was identified by GWAS (273). Since GAK has been found to interact with LRRK2 (274, 275), it may impact its activity. The gene *Rab29*, which encodes Ras-related protein Rab-7L1, has also been linked with risk of Parkinson's disease (273). Rab-7L1 stimulates LRKK2 kinase activity (276) and so may also be involved in regulating LRKK2-induced phosphorylation.

### Ubiquitination

As ubiquitination is a process closely involved in the pathology of Parkinson's disease, numerous enzymes in the ubiquitin pathway have been targeted as potential targets for therapies. Mutations in the *Parkin* gene, encoding an E3 ligase, can lead to familial Parkinson's disease (277, 278). Parkin co-expression with α-synuclein ameliorated α-synuclein toxicity and neuronal loss (279). Co-expression of parkin ameliorated toxicity induced by α-synuclein over expression in substantia nigra neurons of rodents (279). More recently, antibodies were developed to target and inhibit the ubiquitin E3 ligase seven in absentia homologue 1 (SIAH-1). Treatment of cells with the antibodies decreased expression and aggregation of α-synuclein and improved cell viability (280). Knock-down of USP13, a ubiquitin specific protease, in a mouse model mitigated α-synuclein-induced toxicity, as USP13 regulates parkin ubiquitination and therefore indirectly regulates α-synuclein ubiquitination (281).

Furthermore, *PARK6*, is also linked to familial Parkinson's disease (282, 283), and encodes PINK1 (PTEN-induced putative kinase protein 1), which has been shown to phosphorylate and stimulate the E3 ligase activity of parkin (284). The *Park5* and *Park15* genes have also been linked to Parkinson's disease through their involvement in the ubiquitination process. *Park5* encodes a de-ubiquitinating enzyme UCHL1 (285). Protective effects of a UCHL1 variant were observed in a mouse model of Parkinson's disease, indicating this protein may also serve as a target for Parkinson's therapies (286). *Park15* encodes F-box only protein 7 (FBXO7), which functions as an adaptor for an E3 ubiquitin ligase complex (the SKP1/cullin-1/F-box protein), which enables the E3 complex to recognise and ubiquitinate its substrates (287). Loss of function mutations of *Park15* have been identified as causative mutations in familial Parkinson's disease (288), and have been found to colocalise with α-synuclein in Lewy bodies (289). A polymorphism of *Park15* was found to be a protective factor against Parkinson's disease and so targeting FBXO7 may also be relevant for therapeutics (290).

### SUMOylation

SUMOylation may play a role in the intracellular targeting, cellular levels, membrane binding, propagation and aggregation of α-synuclein, and so also could be targeted in the search for a Parkinson's disease therapeutic (165). A recent study examined the effects of α-synuclein SUMOylation, finding that overexpression of a SUMO-conjugase enzyme increased α-synuclein SUMOylation and reduced the toxicity in Parkinson's disease models (291). This finding indicates that increasing SUMOylation of α-synuclein or preventing SUMO removal may be viable targets for Parkinson's disease therapeutics.

### Proteolytic Cleavage and C-Terminal Truncations

Targeting C-terminal truncations could be a viable strategy in Parkinson's disease therapeutic research, as this α-synuclein modification has been observed to be present in Lewy bodies, to accelerate aggregation *in vitro* and *in vivo*, and to enhance prion-like spreading in Parkinson's disease models (124, 125, 208, 292). Antibodies targeting the C-terminus of α-synuclein prevented C-terminal truncation improved Parkinson's pathology and motor symptoms in a mouse model, and reduced propagation of α-synuclein pathology in a cell system (293). Reducing the C-terminal truncation by the pharmacological inhibition of caspase-1, which cleaves α-synuclein at D121, was shown to mitigated neurodegeneration in a transgenic model of multiple system atrophy (294).

### O-GlcNAcylation

O-GlcNAcylation of α-synuclein may offer novel opportunities for the treatment of Parkinson's disease. By building on the observation that O-GlcNAcylation can inhibit α-synuclein aggregation and ameliorate its associated toxicity (128), O-GlcNAcylated α-synuclein peptides from the NAC region were developed and shown to reduce α-synuclein aggregation (295). It has also been proposed that the pharmacological inhibition of O-GlcNAcase can increase the O-GlcNAcylation levels of α-synuclein, resulting in a lower aggregation propensity and in a reduced cellular intake of α-synuclein aggregates (296). O-GlcNAcylation has also been reported to inhibit calpain-mediated C-terminal α-synuclein truncations, which as discussed above increase the aggregation propensity of this protein. Similarly, O-GlcNAcylation competes with phosphorylation in targeting hydroxyl groups on serine and threonine side-chains (127), thus protecting α-synuclein from the increase in aggregation propensity caused by phosphorylation. In a recent study, the pharmacological inhibition of O-GlcNAcase was shown to reduce the accumulation of pS129 α-synuclein in the substantia nigra in a mouse model of Parkinson's disease (297).

## Conclusions

α-Synuclein has been associated with Parkinson's disease over two decades ago (20). This discovery, however, has not yet led to the development of effective diagnostic tools and disease-modifying treatments for this disease. Such a slow progress can be attributed at least in part to the complexity of the structure, function, and interactions of α-synuclein with other cellular constituents.

In particular, the interaction of α-synuclein with cell membranes is important to determine both the function and the dysfunction of this protein. While α-synuclein does not readily spontaneously, lipid membranes provide a surface for the initial nucleation events (15). Following this observation, compounds have been identified that reduce the aggregation of α-synuclein by displacing it from lipid membranes, and reducing oligomer formation in membranes *in vitro*, in primary neuronal cells and in mice models of Parkinson's disease (298, 299).

In this review, we have discussed the role of post-translational modifications of α-synuclein in altering the behaviour of this protein in the presence of lipid membranes (Table 1) (137). N-terminal acetylation has been shown to regulate the binding of α-synuclein to phospholipid membranes, particularly to those of synaptic vesicles (16, 107). Phosphorylation and nitration appear to aggravate the pathology of α-synuclein by decreasing lipid membrane interactions, SUMOylation may be involved in the cell-to-cell spreading of α-synuclein aggregates by enhancing binding to extracellular vesicles, and α-synuclein truncations could promote aggregation through either primary or secondary events (Table 1).

Furthermore, post-translational modifications of α-synuclein can affect and modulate each other. For example, O-GlcNAcylation at S87 was found to regulate phosphorylation of S129 and S87 (126). S87 can be glycated or phosphorylated, K96 can be SUMOylated or ubiquitinated, and K102 can be SUMOylated or the site of truncation. Post-translational modifications might also upregulate other modifications, for example most of the ubiquitinated α-synuclein in Lewy bodies was found to be phosphorylated at S129 (103).

In conclusion, we have described how the investigation of the effects of post-translational modifications on the interaction of α-synuclein with lipid membranes is increasing our understanding the molecular origins of Parkinson's disease, and contributing to the identification of novel targets for therapeutic (253–256) and diagnostic (300, 301) interventions.

## Author Contributions

RB and MV reviewed the literature and wrote the article. Both authors contributed to the article and approved the submitted version.

## Conflict of Interest

The authors declare that the research was conducted in the absence of any commercial or financial relationships that could be construed as a potential conflict of interest.

## Abbreviations

AA: Arachidonic acid
ALA: α-lipoic acid
α-syn: α-synuclein
BOG: β-octyl-glucoside
CD: circular dichroism
Chol: cholesterol
DHA: docosahexaenoic acid 22:6(n-3)
DMPS, 1: 2-dimyristoyl-sn-glycero-3-phospho-L-serine
DAT: dopamine transporter
DOPC, 1: 2-dioleoyl-sn-glycero-3-phosphocholine
DOPE, 1: 2-dioleoyl-sn-glycero-3-phosphoethanolamine
DOPS, 1: 2-dioleoyl-sn-glycero-3-phospho-L-serine
GMPV: giant cell-membrane derived plasma vesicle
LUV: large unilamellar vesicle
L-DOPA, L-3: 4-dihydroxyphenylalanine
MUFA: mono-unsaturated fatty acid
GlcNAc: N-acetylglucosamine
NMR: nuclear magnetic resonance
OGT: O-GlcNAc transferase
OA: oleic acid
PL: phospholipids
PA: phosphatidic acid
PC: phosphatidylcholine
PE: phosphatidylethanolamine
PI: phosphatidylinositol
PS: phosphatidylserine
POPA: 1-palmitoyl-2-oleoyl-sn-glycero-3-phosphate
POPC: 1-palmitoyl-2-oleoyl-glycero-3-phosphocholine
POPG: 1-palmitoyl-2-oleoyl-sn-glycero-3-phospho-(1′-rac-glycerol)
POPS: 1-palmitoyl-2-oleoyl-sn-glycero-3-phospho-L-serine
PUFA: poly-unsaturated fatty acid
PTM: post-translational modification
SUMO: small ubiquitin-like modifier
SUV: small unilamellar vesicle
SDS: sodium dodecyl sulphate
SM: sphingomyelin
SN: substantia nigra
TH: tyrosine hydroxylase
Ub: ubiquitin
VMAT2: vesicular monoamine transporter
WT: wild-type.
